# Simulation of atherosclerotic plaque growth using computational biomechanics and patient-specific data

**DOI:** 10.1038/s41598-020-74583-y

**Published:** 2020-10-15

**Authors:** Dimitrios S. Pleouras, Antonis I. Sakellarios, Panagiota Tsompou, Vassiliki Kigka, Savvas Kyriakidis, Silvia Rocchiccioli, Danilo Neglia, Juhani Knuuti, Gualtiero Pelosi, Lampros K. Michalis, Dimitrios I. Fotiadis

**Affiliations:** 1 Department of Biomedical Research, Institute of Molecular Biology and Biotechnology – FORTH, University Campus of Ioannina, 45110 Ioannina, Greece; 2 Unit of Medical Technology and Intelligent Information Systems, Department of Materials Science and Engineering, University of Ioannina, PO BOX 1186, 45110 Ioannina, Greece; 3 Institute of Clinical Physiology, National Research Council, 56124 Pisa, Italy; 4 Fondazione Toscana G. Monasterio, 56124 Pisa, Italy; 5 Turku PET Centre, University of Turku, and Turku University Hospital, Turku, Finland; 6 Department of Cardiology, Medical School, University of Ioannina, 45110 Ioannina, Greece

**Keywords:** Biomedical engineering, Cardiovascular diseases

## Abstract

Atherosclerosis is the one of the major causes of mortality worldwide, urging the need for prevention strategies. In this work, a novel computational model is developed, which is used for simulation of plaque growth to 94 realistic 3D reconstructed coronary arteries. This model considers several factors of the atherosclerotic process even mechanical factors such as the effect of endothelial shear stress, responsible for the initiation of atherosclerosis, and biological factors such as the accumulation of low and high density lipoproteins (LDL and HDL), monocytes, macrophages, cytokines, nitric oxide and formation of foams cells or proliferation of contractile and synthetic smooth muscle cells (SMCs). The model is validated using the serial imaging of CTCA comparing the simulated geometries with the real follow-up arteries. Additionally, we examine the predictive capability of the model to identify regions prone of disease progression. The results presented good correlation between the simulated lumen area (P < 0.0001), plaque area (P < 0.0001) and plaque burden (P < 0.0001) with the realistic ones. Finally, disease progression is achieved with 80% accuracy with many of the computational results being independent predictors.

## Introduction

According to World Health Organization, coronary heart disease (CHD) is the leading cause of death globally with more than 60% of the global burden to be present in developing countries^1^. The disease is characterized by the reduction of blood supply to the heart muscles by the arteries, which may cause heart failure or death. The biological process that underlies atherosclerotic disease formation is extremely complicated. Nonetheless, a simplified description of this process initiates with the effect of blood rheology and especially the effect of endothelial shear stress (ESS) on the endothelial membrane. This effect may be translated to endothelial dysfunction and increased endothelial permeability to lipid components such as the low density lipoprotein (LDL) particles. LDL is accumulated into the arterial wall and starts being oxidized, triggering an inflammatory response by the accumulation of macrophages and monocytes. Endocytosis of oxidized LDL particles by the inflammatory cells forms foam cells and consequently fatty streaks. In next steps, the smooth muscle cells are proliferated and collagen is produced forming the atherosclerotic plaque^2^.

The above mentioned mechanisms have been attempted to be simulated in several studies. The first published studies focused on the simulation of blood flow and the correlation of ESS with the disease progression, while more complex models simulate the LDL transport in coronary arteries. In recent years, several advanced models have been presented. Most of them are applied on idealized 2D arterial geometries^3,4^, while only a few proof of concept studies are based on realistic human arterial geometries^5–8^.

Nowadays, preventive strategies are considered as the best option to reduce the burden of CHD. These strategies include population based approaches e.g. quit of smoking, exercise, diet, etc. as well as individual based e.g. treatment of hypertension or hyperlipidemia. On the other hand, computational modeling could be used potentially for the development of novel preventive strategies. In this work, we present a novel multi-level computational model for atherosclerotic plaque growth, incorporating the major mechanisms of atherosclerosis. Moreover, it is the first time that such a model has been employed to a dataset of 94 human coronary arteries for its validation. This dataset includes imaging data of computed tomography coronary angiography (CTCA) at two time points with interscan interval of 6.1 years, adequate to assess disease progression.

## Results

**Figure 1:**
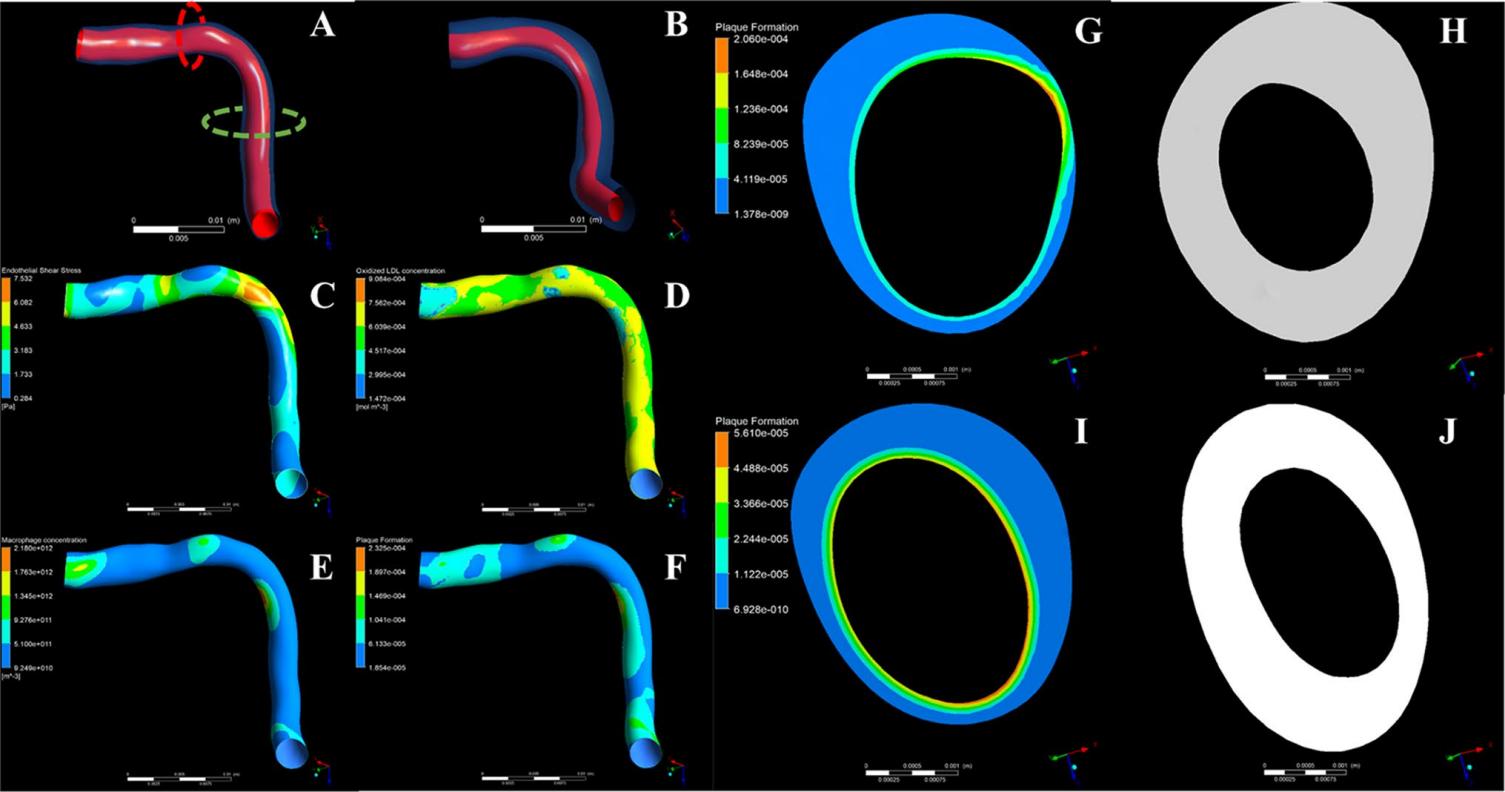
A case example of a coronary artery. (**A**, **B**) show the reconstructed arteries (red: lumen, transparent blue: arterial wall) for baseline and follow-up time point, respectively. The lumen stenosis is presented clearly at the follow-up reconstruction (**B**). (**C**–**F**) Distribution of endothelial shear stress, oxidized LDL concentration accumulation in the arterial wall, macrophages concentration and plaque formation. Regions of low ESS present higher accumulation of oxidized LDL and inflammatory molecules. (**G**, **H**) Cross-section with plaque formation variable at the baseline (**G**) and the corresponding follow-up cross section (**H**). This cross section corresponds at the red dotted line of panel (**A**). (**I**, **J**) Cross-section with plaque formation variable at the baseline (**I**) and the corresponding follow-up cross section (**J**), where this cross section is located at the green dotted line of panel (**A**). (**H**, **J**) Cross-sections of increased plaque area and lumen area reduction as found realistically at the reconstructed arteries.

We performed the plaque growth simulation at 94 coronary arteries, which resulted to 900 3 mm sub-segments. The simulations resulted to the deformed arterial geometries due to plaque growth. The simulation was performed assuming a time period same with the interscan period of each patient applying timesteps of one months. A case example of an arterial segment with increased lumen reduction and plaque increase is shown in Fig. 1. Table 1 presents the mean and Standard Deviation of the lumen and wall area and plaque burden change for the simulated and real arteries as found in the CTCA images.

**Table 1:** The mean and Standard Deviation of lumen and wall area and plaque burden change for the simulated and real arteries.

	Real CTCA based change		Simulated change	
	Mean	Std. deviation	Mean	Std. deviation
Lumen area change	− 0.72	2.58	− 1.16	2.21
Wall area change	1.13	3.59	2.21	2.37
Plaque burden change	0.94	16.95	4.79	13.21

**Figure 2:**
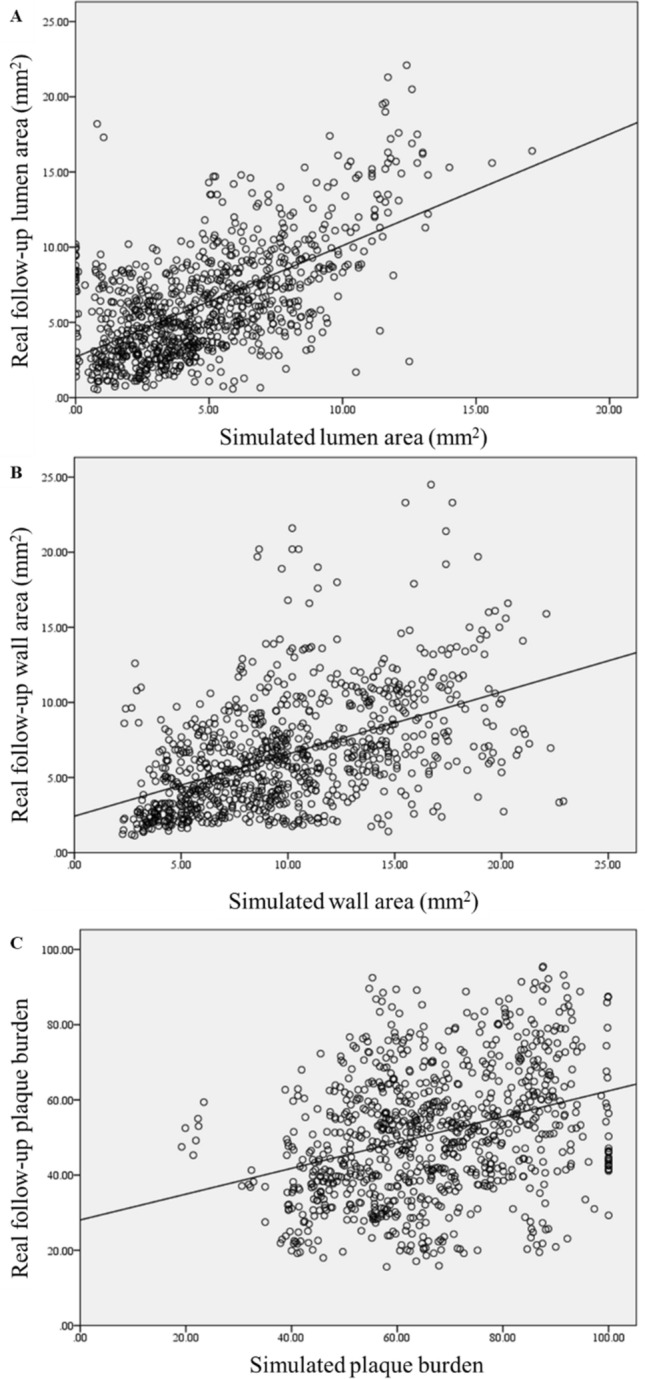
Scatter dot plots between the simulated findings with the real follow-up for the lumen area, wall area and plaque burden.

Among the results, all species’ distributions within the arterial wall were extracted in all time steps of the simulations, in order to further demonstrate each patient’s arterial wall composition over time. The comparison of the simulated and the real follow-up arterial lumen area showed a statistical significant correlation (r = 0.608, P < 0.0001). Similar correlation is also found between the simulated wall area with the corresponding real follow-up area (r = 0.494, P < 0.0001). Finally, the simulated plaque burden is also associated with the real follow-up plaque burden, presenting, however, lower correlation coefficient (r = 0.332, P < 0.0001). These correlations are also presented in Fig. 2 in the form of scatter plot.

**Figure 3:**
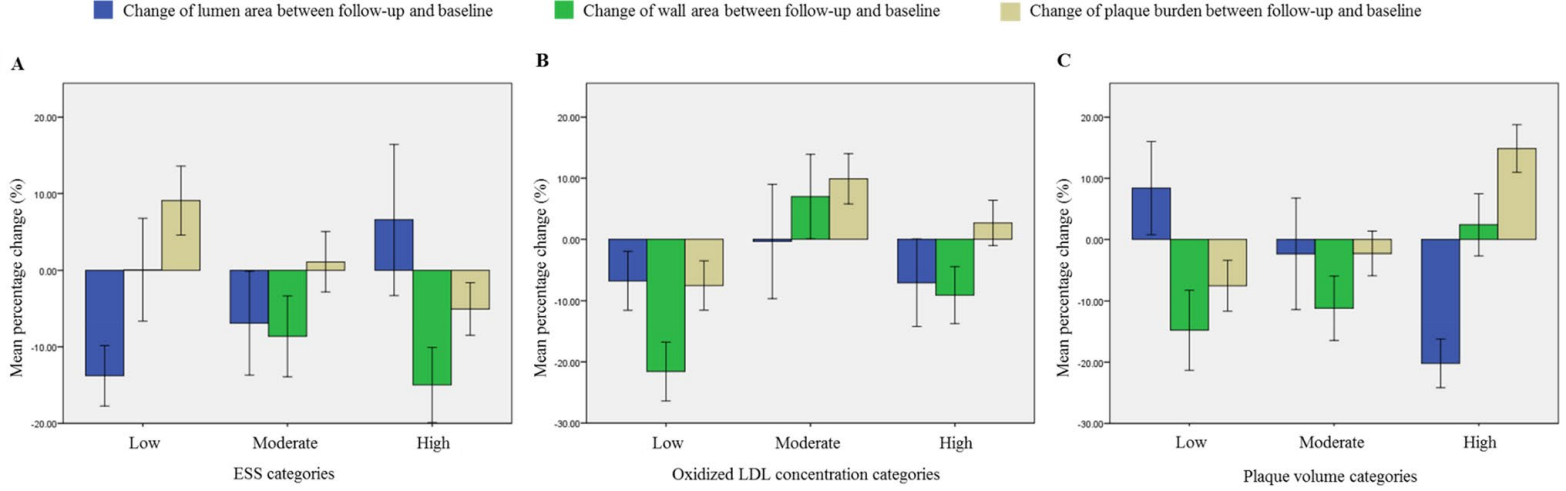
Association between endothelial shear stress (ESS) and oxidized LDL concentration and plaque volume categories with the local change in lumen area (blue), wall area (green) and plaque burden (yellow) between the follow-up and the baseline.

Going one step further, we hypothesize that the plaque growth model and its variables could be further used for the prediction of disease progression. For this purpose, we estimate the disease progression between the follow-up and the baseline for each 3 mm sub-segment. Initially, linear regression analysis was performed to identify correlations between the computational variables and the lumen area, plaque area and plaque burden change. We found a strong relation of ESS with disease progression, since regions of low ESS present increased lumen area reduction (P < 0.0001) and an increase of the plaque area and plaque burden at the follow-up (P < 0.0001). This is explained by the fact that in areas of high shear stress, blood velocity is increased preventing several species from entering in the arterial wall, as they drift along with blood. This mainly occurs in cells which due to their large size maintain their momentum, drifting along with blood. The arterial wall concentrations of foam cells, synthetic smooth muscle cells and collagen have a direct association to the plaque volume, as it is evaluated based on their volume. Figure 3 presents the relation of some computational variables with the change of lumen area, plaque area and plaque burden.

A multi-variate linear regression model was also built. Collinear variables (> 0.8 collinearity) were not included in the statistical model. Regarding the lumen area change, the included variables were the baseline plaque burden (B = 0.048, P < 0.0001), the collagen concentration (B = − 79.26, P < 0.0001) and the HDL concentration (B = 1538.00, P < 0.0001). The associated variables regarding the prediction of wall area change are the baseline lumen area (B = 0.16, P < 0.0001) and baseline wall area (B = − 0.52, P < 0.0001), the monocytes concentration (B = 1.99 × 10^–9^, P = 0.003), the macrophages concentration (B = − 1.07 × 10^–11^, P < 0.0001), the oxidized LDL concentration (B = 2441.94, P < 0.0001), the HDL concentration (B = − 3411.139, P < 0.0001) and the ESS (B = − 0.16, P = 0.014). Finally, regarding the plaque burden change, the associated variables are baseline plaque burden (B = − 0.45, P < 0.0001), the monocytes concentration (B = 2.48 × 10^–8^, P < 0.0001), the cytokines concentration (B = − 1.97, P < 0.0001), the oxidized LDL concentration (B = 11,967.47, P < 0.0001), the HDL concentration (B = − 0.11, P = 0.002), baseline lumen area (B = − 0.62, P = 0.001) and the collagen concentration (B = 195.03, P = 0.036).

Finally, the prognostic value of the plaque growth model in identifying segments of disease progression was achieved performing binary logistic regression modeling. To this purpose, we first performed a receiver-operator characteristics analysis to identify cut-off points to make binary the associated variables identified in the linear multi-variate analysis. Lumen area change, plaque area change, and plaque burden change were transformed to binary assuming 20% change. The accuracy to predict lumen reduction, plaque increase, and plaque burden increase is 83%, 80% and 77%, respectively.

## Discussion

In this work, we have developed a new plaque growth model which simulates the blood flow dynamics, the species transport in the arterial wall, the oxidation of LDL, the inflammation, the formation of foam cells and finally the development and growth of plaque consisted of smooth muscle cells, collagen and foam cells. Specifically, this model introduces each one of these features in one of its three modelling levels: (i) blood flow modelling and evaluation of ESS distribution, (ii) lipoprotein (LDL, and HDL) transport within the arterial wall, inflammation modelling and plaque volume evaluation, (iii) wall thickening modelling. It is the first time that a complex model has been applied to a population with serial imaging-based assessment to validate its outcomes. More interestingly, the selected population is considered low-risk, since no invasive angiography is available for these patients. Moreover, for the first time, prediction of disease progression is achieved with 80% accuracy using computational results other than the ESS and the LDL concentration.

A significant characteristic of this model is that the endothelial dysfunction was integrated in the endothelial permeability model. Endothelial dysfunction is the culprit of atherosclerosis initiation, while some major influential factors leading to endothelial dysfunction are the ESS magnitude and the endothelial nitric oxide concentration level, where both of them are included in this model. More specifically, low ESS is experimentally found to promote the endothelial fluxes of lipoproteins, a fact that is explained due to the increase of the endothelium intercellular space.

Increased lipoproteins accumulation within the arterial wall triggers the inflammatory process initiated with the oxidation of LDL caused by free radicals. Subsequently, the presence of OxLDL within the arterial wall causes cytokine production, which is an inflammatory signalling molecule for monocyte attraction. Therefore, monocytes accumulate within the affected area and differentiate into macrophages to uptake the OxLDL. The presence of clusters with foam cells in areas containing abnormal lipoprotein concentrations was established by histological analyses, ultimately exposing type I lesions. Increased concentrations of lipid-laden smooth muscle cells were detected in advanced lesions, leading to the classification of type II lesions, namely fatty streaks. Extracellular lipid particles can be encountered in further advanced lesions, preventing the coherence of smooth muscle cells in the intima layer, eventually leading to type III and IV lesions, distinguished by the size of the extracellular lipid droplets. Type V lesions, distinguished by the existence of thick layers of fibrous connective tissues in the lesions, prevail due to additional advancement of the latter lesion classes, typically occurring during the fourth decade of life. Lastly, manifestation of fissure, hematoma or thrombus within the lesion, led to their referral type IV lesions. Our model considers the dynamics of LDL, foam cells and smooth muscle cells within the arterial wall and therefore it constitutes a mathematical tool for predicting type I and II lesions. A novelty of this work is that the wall thickening is enabled in the atherosclerotic areas and it is based on the total calculated plaque volume, which consists of foam cells, collagen and smooth muscle cells—the main components of the fatty streak plaques. These components that can identify both type I and II lesions, are used to evaluate the volumetric growth of the arterial wall, by considering the volumetric growth in each element of the arterial wall. This is important for development of prevention strategies. In fact, the selected population of SMARTool project consists of low-risk patients without events. For this reason, the developed plaque growth model can be used to predict the arterial progress of patients with at least 20% disease progression in a period of six years.

A major novelty of this work is the use of a low-risk population with a large follow-up. This enables the examination of disease progression to patients who are not susceptible to disease progression compared to interventional studies, which include high-risk patients. Similar models were presented previously^6,9^, but only as proof-of-concept approaches or in ideal geometries. Our work, however, can be compared against other predictive studies of disease progression, which are mainly using ESS and LDL concentration accumulation as predictors^5,10,11^. In fact, in these studies, maximum accuracy of previous studies to predict disease progression is less than 65%. Our results show that in our population we have about 80% accuracy of disease progression defined as lumen reduction, plaque increase or plaque burden increase. This contributes to the conclusion that this complex computational model may describe better the pathophysiology of atherosclerosis and worth applied to even larger populations.

However, the main outcome of this work is the dynamics of this proof of concept study. Compared to ESS based predictive models, where only blood flow modeling is implemented, using a plaque growth model the pathophysiology of the disease is simulated. In this concept, it is possible to increase the complexity of the model by the inclusion of additional biological pathways. Considering large initiatives for data collection including omics data, a plaque growth model can be improved considerably adding the effect of genetic phenotype or implementing machine learning approaches for the prediction of disease progression.

The current work has however some limitations. First, the population even if it is the largest population used for the validation of such models, it is considered still small to make safe conclusions. However, the results demonstrate the trend that this model should be applied to larger populations and potentially can be used as a preventive tool. Also, the different stiffness of the plaque regions was neglected, since almost all patients presented low disease progression and small plaque regions. Finally, in this work, we didn’t consider the plaque composition and the relation of the computational variables with specific plaque types. This task requires the use of intravascular imaging either ultrasound or optical coherence tomography. We aim to utilize such datasets in a future work.

## Conclusions

We have developed and validated a multi-level plaque growth model using serial CTCA imaging from 94 patients. The plaque growth model simulates the main mechanisms of disease progression. The results show that the simulated and generated arterial geometries are correlated well with the real follow-up geometries. Additionally, we investigated the role of the plaque growth model to predict disease progression. Our results show that prediction of lumen area reduction and plaque area increase can be achieved with 80% accuracy.

## Methods

### Population, CTCA analysis and 3D reconstruction

CTCA imaging was acquired from the SMARTool clinical study, which is a multi-center EU funded (GA number: 689068) project aiming to the development of decision support systems for the management of CHD in terms of risk stratification, diagnosis and prognosis. Each patient’s dataset included baseline clinical and biohumoral data and CTCA imaging at two time points. In the current analysis, we have used only the LDL and HDL concentration as boundary condition, the blood pressure applied in the equations and the interscan period of each patient to define the maximum simulation period. The clinical characteristics of our population is presented in Table 2. For the present analysis 94 patients were selected. More specifically, a population of 275 patients was created in SMARTool project, from which 12 patients did not perform follow-up CTCA and 76 were excluded due to stenting after the baseline examination or low image quality. This results at 187 patients, from which we randomly selected 50% of them (94 patients) from various clinical centers. Full explanation of the investigational nature of the study was provided to all participants and written consensus obtained. Ethical approval was provided by each participating center (National Research Council, University of Turku, University of Zurich, Fondazione Toscana Gabriele Monasterio, Warsaw National Institute of Cardiology) through the approval of the clinical study by the Ethics Committee Vast Area Northwest of Tuscany (CEAVNO), Pisa, Italy, and all subjects gave written informed consent. Our clinical study follows the declaration of Helsinki.

3D reconstruction of the coronary lumen and outer vessel wall was performed using an in-house software, which provided measurements of lumen area, plaque volume and plaque burden as previously described and validated^12,13^. Finally, baseline and follow-up CTCA scans were co-registered using landmarks such as the bifurcations and calcified objects.

**Table 2:** Clinical characteristics of our population.

	All patients (N = 94)
Age at follow-up (years)	60.30 ± 8.54
Interscan period (years)	6.11 ± 1.34
Gender (male)	57 (60.64%)
Current smoker	13 (13.83%)
Family history of CAD	49 (52.13%)
**Co-morbidities**	
Diabetes mellitus	14 (14.89%)
Hypertension	116 (58.29%)
Dyslipidemia	68 (72.34%)
Obesity	20 (21.28%)
**Biochemical**	
Triglycerides mg/dL	113.81 ± 62.92
Total cholesterol mg/dL	185.55 ± 45.63
LDL mg/dL	108.16 ± 38.08
HDL mg/dL	55.96 ± 15.51
**Medications at discharge**	
Aspirin	51 (54.26%)
ARB	13 (13.83%)
Beta-blocker	37 (39.36%)
ACE inhibitors	23 (24.47%)
Statin	44 (46.81%)
**Stenosis at baseline**	
No stenosis	17 (18.09%)
< 30%	17 (36.17%)
30–50%	21 (22.34%)
50–70%	13 (13.83%)
70–90%	6 (6.38%)
> 90%	3 (3.19%)

*LDL* low density lipoprotein, *HDL* high density lipoprotein, *ARB* Angiotensin II Receptor Blockers, *ACE* Angiotensin-converting enzyme.

### Multi-level plaque growth model

The developed plaque growth model consists of three modeling levels: (i) the steady stae simulation of blood flow, (ii) the transient simulation of the LDL, HDL and monocytes accumulation within the arterial wall and the atherosclerotic plaque generation, resulting in the estimation of plaque volume and (iii) the simulation of plaque growth in time. The simulation of the blood flow results in the evaluation of the endothelial shear stress distribution, which is necessary for the evaluation of the trans-endothelial flow rates. Even though the arterial wall thickens over time due to atherosclerosis, we assume that the transient change of the lumen geometry does not affect the wall shear stress distribution significantly. The transient simulation of the atherosclerotic process results in the evaluation of the plaque density distribution. The resulted plaque density distribution of the last timestep is then used to evaluate the volumetric strain and enable the simulation of the arterial wall thickening. The mathematical formulation is based on differential equations, which are presented in detail in the Appendix.

#### Blood flow dynamics

Our model considers blood flow in the lumen domain and a plasma flow in the arterial wall domain. Blood is considered as an incompressible Newtonian fluid that presents a density value of 1060 [kg/m^3^]^14^ and a dynamic viscosity value of 0.0035 [Pa s]^15^. The fluid dynamics of blood is based on the Navier–Stokes equations for laminar, incompressible and Newtonian flows, that account for momentum and continuity conservation, respectively. A steady blood flow was assumed, since this model considers a time duration of years and therefore a steady flow profile is assumed that does not affect the plaque growth (1–3).

1 $$\nabla \cdot (\uprho \mathbf{U})=0,$$

2 $$\nabla \cdot \left(\uprho \mathbf{U}\otimes \mathbf{U}\right)=-\nabla \mathrm{p}+\nabla \cdot\uptau ,$$

3 $$\mathrm{where},\uptau =-{\upmu }_{\mathrm{blood}}\left(\nabla \mathbf{U}+{\left(\nabla \mathbf{U}\right)}^{\mathrm{\rm T}}-\frac{2}{3}\updelta \nabla \cdot \mathbf{U}\right).$$

*ρ* is the blood density, *U* is the blood velocity, *P* is the pressure, *τ* is the shear stress, *μ*_*blood*_ is the blood’s dynamic viscosity and *S*_*M*_ accounts for momentum sources.

Plasma is considered as an incompressible Newtonian fluid that presents a density value of 1000 [kg/m^3^]^14^ and a dynamic viscosity value of 0.001 [Pa s]^15^. Plasma flow in the arterial wall is governed by the modified Navier–Stokes equations laminar, incompressible and Newtonian flows inside a porous media, since the arterial wall can be characterized as such, with a porosity coefficient 0.96^16^. These equations derive from the Navier–Stokes and continuity equations, in which the reduced flow space is considered using the area porosity tensor ***K*** while the momentum losses are considered in a source term *S*_*M*_. In a material of porosity *γ*, the area porosity tensor is calculated by (4). The momentum losses *S*_*M*_ are formulated using the permeability *K*_*perm*_ and loss coefficient *K*_*loss*_, but in our case of a low plasma velocity within the arterial wall, the term including the loss coefficient *K*_*loss*_ is neglected as it includes a second degree of the velocity that is tiddley (8). Using the porosity *γ* and the area porosity tensor ***K***, the equations for the conservation of mass (5) and momentum (6) account for the decreased flow space and the momentum losses^17,18^.

4 $${\mathrm{K}}^{\mathrm{ij}}=\upgamma {\updelta }^{\mathrm{ij}},$$

5 $$\frac{\partial }{\partial \mathrm{t}}\mathrm{\gamma \rho }+\nabla \cdot (\uprho \mathbf{\rm K}\cdot \mathbf{U})=0,$$

6 $$\frac{\partial }{\partial \mathrm{t}}\left(\mathrm{\rho \gamma }\mathbf{U}\right)+\nabla \left(\uprho \left(\mathbf{K}\cdot \mathbf{U}\right)\otimes \mathbf{U}\right)-\nabla \cdot\uptau =-\upgamma \nabla \mathrm{p}+\upgamma {\mathrm{S}}_{\mathrm{M}},$$

7 $$\mathrm{where},\uptau =-{\upmu }_{\mathrm{plasma}}\mathbf{K}\left(\nabla \mathbf{U}+{\left(\nabla \mathbf{U}\right)}^{\mathrm{\rm T}}-\frac{2}{3}\updelta \nabla \cdot \mathbf{U}\right),$$

8 $${\mathbf{S}}_{\mathrm{M},\mathrm{i}}= -\frac{\upmu }{{\mathrm{K}}_{\mathrm{perm}}}{\mathrm{U}}_{\mathrm{i}}.$$

*μ*_*plasma*_ is the plasma viscosity, *K*_*perm*_ is the Darcian permeability, *γ* is the volume porosity and *K* is the area porosity tensor, which is a symmetric second rank tensor. Momentum losses, *S*_*M*_, account for the losses due to the permeability decrease and the velocity direction changes^17,18^.

#### Dynamics of the atherosclerotic process

In lumen, blood flows along with LDL, HDL and monocytes. The blood concentration of LDL, HDL and monocytes are patient specific and therefore are used as boundary conditions. However, after their infiltration in the arterial wall, their concentrations within the arterial wall are calculated using the convection–diffusion-reaction Eq. (9), which is also modified to account for flow in porous media as presented previously^18,19^.

9 $$\frac{\partial }{\partial t}\left(\gamma c\right)+\nabla \cdot \left({\varvec{K}}\cdot {\varvec{U}}c\right)=\nabla \cdot \left(D{\varvec{K}}\cdot \nabla c\right)+\mathrm{S}c.$$

The first term accounts for time-dependent concentration changes, while the second is the advection term and accounts for concentration changes due to velocity drift. The third one is the diffusion term, where *D* is the diffusivity. The last term accounts for concentration sources or sinks.

LDL macromolecules react with the free radicals in the arterial wall, forming oxidized LDL and therefore their concentration reduces^20^. However, HDL macromolecules, that infiltrate to the arterial wall, also react with the free radicals and reduce the free radical concentration, leading to a decrease of the LDL oxidation rate. An HDL-dependent oxidation rate of LDL was implemented as it was presented by Sakellarios et al.^6^ which was based on the experimental results of Esterbauer et al.^20^, and it is referred as *HDL*_*protection*_.

10 $$ \frac{\partial }{\partial t}\left( {\gamma c_{LDL} } \right) + \nabla \cdot \left( {{\varvec{K}} \cdot {\varvec{U}}c_{LDL} } \right) = \nabla \cdot \left( {D_{LDL} {\varvec{K}} \cdot \nabla c_{LDL} } \right) - {\upgamma }HDL_{protection} r_{LDL} c_{LDL} , $$

11 $$ \frac{\partial }{\partial t}\left( {\gamma c_{HDL} } \right) + \nabla \cdot \left( {{\varvec{K}} \cdot {\varvec{U}}c_{HDL} } \right) = \nabla \cdot \left( {D_{LDL} {\varvec{K}} \cdot \nabla c_{HDL} } \right) - {\upgamma }HDL_{protection} r_{HDL} c_{HDL} . $$

In the LDL sink term, *r*_*LDL*_ (= 1.4 × 10–4 s^−1^^20^) is the LDL oxidation rate, in the absence of HDL macromolecules, while *HDL*_*protection*_ (= − 3 × 10^−5^c_LDL_^2^ + 5 × 10^–4^ c_LDL_ + 1.0056^6^) corresponds to the relative decrease of the LDL oxidation rate due to the presence of HDL macromolecule.

The presence of oxidized LDL (OxLDL) within the arterial wall sets an inflammatory signalling to monocytes, which are accumulated within the arterial wall and differentiate into macrophages to uptake OxLDL, forming foam cells.

12 $$ \frac{\partial }{\partial t}\left( {\gamma c_{OxLDL} } \right) + \nabla \cdot \left( {{\varvec{K}} \cdot {\varvec{U}}c_{OxLDL} } \right) = \nabla \cdot \left( {D_{OxLDL} {\varvec{K}} \cdot \nabla c_{OxLDL} } \right) + \gamma r_{LDL} c_{LDL} - \gamma k_{2} c_{OxLDL} c_{M} . $$

*k*_*2*_ is the OxLDL uptake rate from monocytes, (= 12 × 10^−19^m^3^ cells^−1^ s^−1^^21^) and depends on both OxLDL and macrophage concentration.

Each equation describing a cells’ dynamics has a zero term for advection, due to the cell’s large size in respect to the porous dimension. In most of them the diffusion term is also neglected for the same reason. However, the diffusion terms of monocyte and macrophage dynamics are considered, as we assume that these immune cells can move according to diffusion forces as previously presented by Cilla et al.^9^.

13 $$\frac{\partial }{\partial \mathrm{t}}\left(\upgamma {\mathrm{c}}_{\mathrm{monocytes}}\right)=\nabla \cdot \left({\mathrm{D}}_{\mathrm{m}}\mathbf{K}\cdot \nabla {\mathrm{c}}_{\mathrm{monocytes}}\right)-\upgamma {\mathrm{m}}_{\mathrm{d}}{{\mathrm{c}}_{\mathrm{monocytes}}-\mathrm{\gamma d}}_{\mathrm{m}}{\mathrm{c}}_{\mathrm{monocytes}}.$$

Monocyte apoptosis rate is *m*_*d*_ (= 2.572 cells s^−1^), while *d*_*m*_ (= 1.15 × 10^–6^ s^−1^^22^) is the monocyte differentiation rate to macrophages.

14 $$ \frac{\partial }{\partial t}\left( {\gamma c_{Macrophages} } \right) = \nabla \cdot \left( {D_{M} {\varvec{K}} \cdot \nabla c_{Macrophages} } \right) + \gamma d_{m} c_{monocytes} - \gamma k_{1} c_{OxLDL} c_{Macrophages} , $$

15 $$\frac{\partial }{\partial t}\left(\gamma {c}_{Foam cells}\right)=\gamma {k}_{1}{c}_{OxLDL}{c}_{Macrophages}$$

The macrophages differentiation rate to foam cells is *k*_*1*_ (= 3.671 × 10^–6^ m^3^mol^−1^ s^−1^^9^) and it depends on both the OxLDL and macrophage concentration.

Cytokines are produced due to the presence of macrophages and OxLDL within the arterial wall and cause the differentiation of contractile smooth muscle cells (SMCs) into synthetic SMCs.

16 $$ \frac{\partial }{\partial t}\left( {\gamma c_{cytokines} } \right) = - \gamma d_{c} c_{cytokines} + \gamma d_{r} c_{OxLDL} c_{Macrophages} , $$

17 $$ \frac{\partial }{\partial t}\left( {\gamma c_{Contractile SMCs} } \right) = - {\upgamma }\left( {1 + e^{{\frac{{ - S_{r} c_{cytokines} }}{{c_{{c,{ }max}} }}}} } \right)c_{Contractile SMCs} , $$

18 $$ \frac{\partial }{\partial t}\left( {\gamma c_{Synthetic SMCs} } \right) = {\upgamma }\left( {1 + e^{{\frac{{ - S_{r} c_{cytokines} }}{{c_{c, max} }}}} } \right)c_{ContractileSMCs} . $$

The cytokine degradation rate is *d*_*c*_ (= 2.3148 × 10^–5^ s^−1^^23^) and its production rate is *d*_*r*_ that depends on the OxLDL and macrophage concentration. The contractile SMCs differentiation rate into synthetic SMCs has been found experimentally to be equal to (1 + exp(− *S*_*r*_* c*_*cytokines*_/*c*_*cytokines,max*_)), where *S*_*r*_ = 4.16 × 10^–8^ s^−1^^24^, showing its explicit dependence to the cytokine concentration.

Synthetic SMCs secrete collagen as connective tissue, further increasing plaque volume.

19 $$ \frac{\partial }{\partial t}\left( {\rho \gamma c_{collagen} } \right) = \gamma g_{r} c_{Synthetic SMCs} - \gamma d_{g} c_{collagen} . $$

The collagen production rate from synthetic SMCs is *g*_*r*_ (= 2.157 × 10^–11^ g cells^−1^ s^−1^^25^) while its degradation rate is *d*_*g*_ (= 3.85 × 10^–7^ s^−1^^26^).

#### Endothelial flow rates

The infiltration of species and cells through the endothelium layer can be performed by three different pathways. These are the vesicular transcytosis pathway and the pathways through leaky and normal junctions. The first one accounts for intercellular transport, which is only active in the presence of endothelial cell receptors^27^. The other two, account for transport between the endothelium cells, which are regulated only by mechanical and diffusion factors, such as pressure and concentration differences across the endothelium. The volume fluxes of vesicular transcytosis are negligible in relation to the other two, which allows the application of the Kedem–Katchalsky equations that calculate fluxes based on pressure and concentration differences across biological membranes^28^. The endothelial monocyte fluxes initiate after inflammatory signalling and are defined by an experimental equation using both the OxLDL concentration and the blood monocyte concentration, but also the endothelial ESS. In particular, high ESS retain monocyte from infiltration.

Nitric oxide concentration is considered implicitly in the LDL and HDL diffusive permeability. It is a chemical product of the endothelial nitric oxide synthases (eNOS) and its concentration depends on the partial pressure of oxygen and the eNOS concentration^29^. Following, eNOS concentration dependence of the applied endothelial WSS has been proven experimentally^30^.

20 $${\mathrm{J}}_{\mathrm{V}}={\mathrm{L}}_{\mathrm{p}}\mathrm{\Delta p},$$

21 $${\mathrm{J}}_{\mathrm{S},\mathrm{LDL}}={\mathrm{DP}}_{\mathrm{LDL}}\mathrm{\Delta c}+\left(1-{\upsigma }_{\mathrm{f}}\right)\mathrm{U}\stackrel{-}{\mathrm{c}},$$

22 $${\mathrm{J}}_{\mathrm{S},\mathrm{HDL}}={\mathrm{DP}}_{\mathrm{HDL}}\mathrm{\Delta c}+\left(1-{\upsigma }_{\mathrm{f}}\right)\mathrm{U}\stackrel{-}{\mathrm{c},}$$

23 $${\mathrm{J}}_{\mathrm{monocytes}}=\frac{{\mathrm{m}}_{\mathrm{r}}}{1+(\mathrm{WSS}/{\mathrm{WSS}}_{0})}{\mathrm{c}}_{\mathrm{OxLDL}}{\mathrm{c}}_{\mathrm{monocytes}, \mathrm{lumen}}.$$

Equation (21) accounts for plasma velocity through the endothelium, while Eqs. (22–24) account for LDL, HDL and monocyte fluxes, respectively. The second term of the plasma velocity equation, which corresponds to the decrease of velocity due to osmotic pressure differences, is neglected. *L*_*p*_ is the hydraulic conductivity of the endothelium and it is a function of the endothelial ESS developed from blood flow. *DP*_*LDL*_ and *DP*_*HDL*_ are the LDL and HDL diffusive permeability respectively, and are a function of the endothelial nitric oxide concentration (NO)^4^. In the equation of monocyte flux, *m*_*r*_ (= 5.5 × 10^–4^ m^4^ mol^−1^ day^−1^^9,31^) and ${ESS}_{0}$ (= 1 Pa) are constant values, that derive from experimental data.

24 $${\mathrm{L}}_{\mathrm{P}}=\left(0.2077\mathrm{x}{10}^{-12}\mathrm{ln}\left(\mathrm{WSS}+0.015\right)+3.1588\mathrm{x}{10}^{-12}\right),$$

25 $${\mathrm{DP}}_{\mathrm{LDL}}={\mathrm{DP}}_{\mathrm{HDL}}=\left(-6{ \mathrm{c}}_{\mathrm{NO}}+0.34\right)$$

Endothelial NO concentration depends on the partial pressure of oxygen and the endothelial nitric oxide synthase concentration (eNOS), which is a function of the mean applied endothelial shear stresses^30^.

26 $${\mathrm{c}}_{\mathrm{NO}}={\mathrm{c}}_{\mathrm{NO}, \mathrm{max}}\left(\frac{{\mathrm{p}}_{{\mathrm{O}}_{2}}}{{\mathrm{p}}_{{\mathrm{O}}_{2}}+{\mathrm{k}}_{\mathrm{M}}}\right),$$

27 $${\mathrm{c}}_{\mathrm{NO},\mathrm{max}}={1.26 \mathrm{c}}_{\mathrm{eNOS}},$$

28 $${\mathrm{c}}_{\mathrm{eNOS}}=\left(0.0033 ln\left(\mathrm{WSS} \right)+0.0322\right).$$

#### Wall thickening

To simulate the arterial wall thickening, the arterial wall is treated as a solid domain, which expands due to the volumetric strain that is caused by the generation of plaque. More specifically, the volumetric strain is calculated based on the simulated plaque volume, which enables a strain-based arterial wall thickening. Volumetric strain is defined as the change in volume divided by the original volume^32^. Although, the most used stress–strain relationship of the arterial wall is a 6-parameter Mooney–Rivlin material model^33,34^, we implemented a linear elastic material model, since the volumetric strains that are caused due to plaque growth are relatively small. According to that the arterial wall presents a young modulus of 1.06 MPa and a Poisson ratio of 0.45^7^.

Due to the low plaque progression rate of our population, we assumed that a one-way interaction between the CFD analysis of the atherosclerotic process and the structural analysis of the wall thickening is adequate. Therefore, in our analysis, the volume change of each finite element is equal to the total volume of the foam cells, collagen and synthetic muscle cells, because their initial concentration inside the arterial wall was considered zero.

29 $$ Ratio_{{\frac{Plaque volume}{{Finite Volume}}}} = \frac{\partial V}{V} = c_{Foam cells} *V_{Foam cell} + c_{Synthetic SMC} *V_{Synthetic SMC} + c_{collagen} *v_{collagen} , $$

30 $$\frac{\partial \mathrm{V}}{\mathrm{V}}=\left(1+{\upvarepsilon }_{\mathrm{xx}}\right)\left(1+{\upvarepsilon }_{\mathrm{yy}}\right)\left(1+{\upvarepsilon }_{\mathrm{zz}}\right)-1\Rightarrow \frac{\partial \mathrm{V}}{\mathrm{V}}\approx {\upvarepsilon }_{\mathrm{xx}}+{\upvarepsilon }_{\mathrm{yy}}+{\upvarepsilon }_{\mathrm{zz}}.$$

where, in (11), *c*_*Foam cells*_, *c*_*Synthetic SMC*_ and *c*_*collagen*_ are the concentrations of foam cells (cells/m^3^), synthetic SMCs (cells/m^3^) and collagen (g/m^3^) respectively, while *V*_*Foam cells*_, *V*_*Synthetic SMC*_ and *v*_*collagen*_ are the cellular volume of foam cells and the cellular volume of synthetic SMCs and the specific volume of collagen respectively. Sequentially, in (30), *V* is the volume of a cubic finite element and *ε* is the resulted strain after a change in volume ∂*V*. In our model, we consider a constrained arterial wall thickening that is available only to the direction of the centerline and therefore the directional volumetric strain *ε*_*directional*_ can result from the function:

31 $$\frac{\partial \mathrm{V}}{\mathrm{V}}=\frac{(\mathrm{l}+\mathrm{dl}){\mathrm{l}}^{2}-{\mathrm{l}}^{3}}{{\mathrm{l}}^{3}}=\frac{\mathrm{dl}}{\mathrm{l}}={\upvarepsilon }_{\mathrm{directional}}.$$

#### Numerical implementation

The simulations were conducted using the ANSYS software (ANSYS, Canonsburg, PA) which integrate among other finite element and finite volume analysis solvers. Specifically, fluid dynamics are performed using the ANSYS CFX software, while the wall thickening is performed in ANSYS mechanical module.

**Figure 4:**
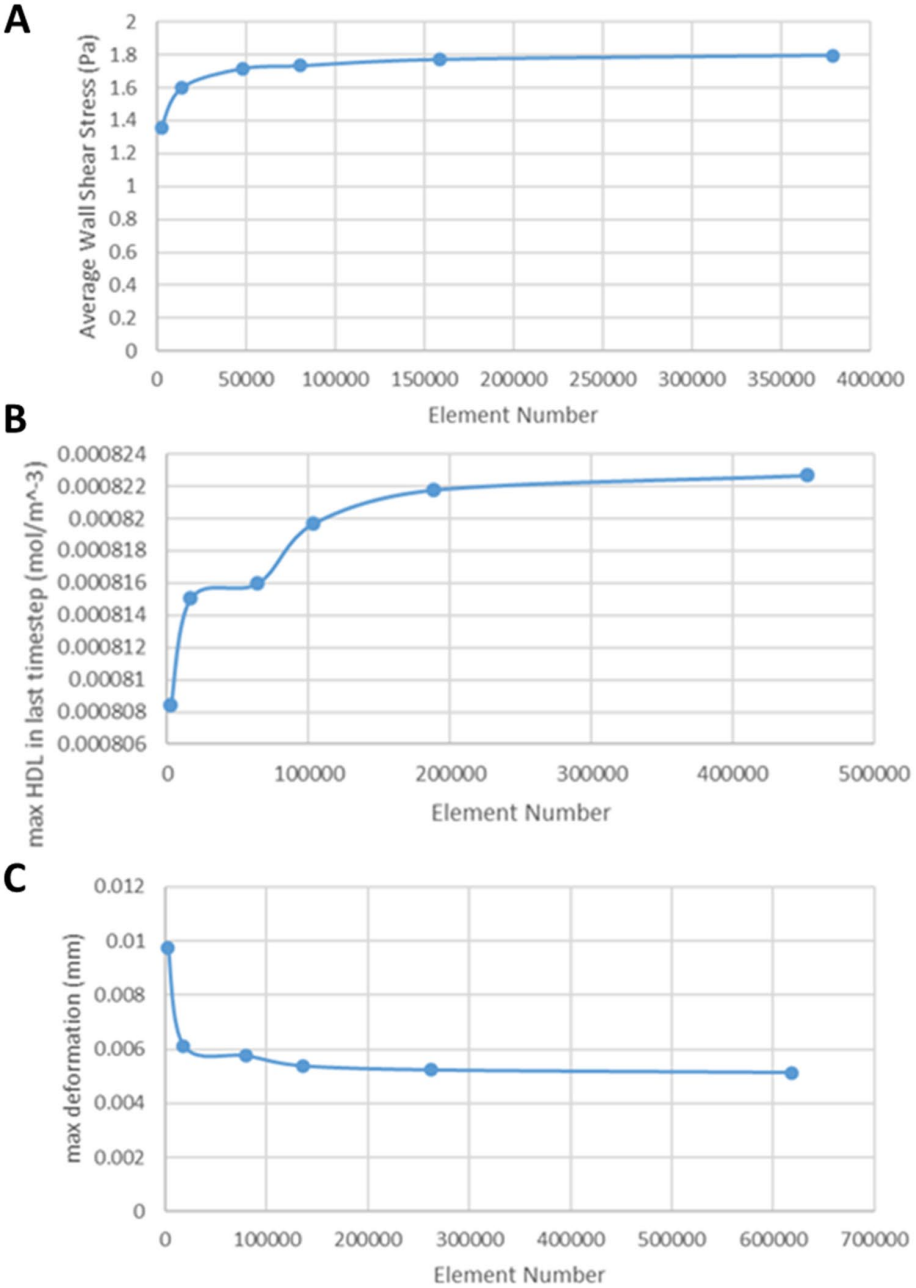
(**A**) Mesh refinement study for the blood flow analysis, (**B**) Mesh refinement study for the analysis of the atherosclerotic process, (**C**) Mesh refinement study for the wall thickening analysis.

Both lumen and the arterial wall domain of each patient were meshed using tetrahedral elements of 0.15 mm edges. Several methods were used to improve the mesh quality, such as the patch-independent technique and the inflation. Specifically, these parameters were evaluated after monitoring of the element quality statistics. Moreover, a sensitivity analysis of a normal/typical patient arterial segment was performed based on the tetrahedral element size, to further validate the mesh quality (Fig. 4).

### Boundary conditions

#### Fluid domain of lumen

To simulate the blood flow in lumen, inlet is conditioned by the patient blood velocity, outlet is conditioned by the patient blood pressure and the endothelium layer is considered as an impenetrable and no-slip wall, since endothelial fluxes are too small to affect the blood flow.

#### Fluid domain of arterial wall

Plasma flow in the arterial wall is constrained with a zero-velocity to the vertical sides of the arterial wall, a standard pressure to the adventitia layer and a Kedem-Katchalsky derived velocity at endothelium. The arterial wall vertical sides are impenetrable to any substance and species, while at the adventitia layer all substances can pass through. The adventitia layer is also constrained with patient-specific LDL and HDL concentration values.

#### Solid domain of arterial wall

Regarding the arterial wall thickening, endothelium’s displacement is enabled only in the direction towards the arterial centerline, while in the adventitia and the arterial wall sides are conditioned with a frictionless support.

### Validation

**Figure 5:**
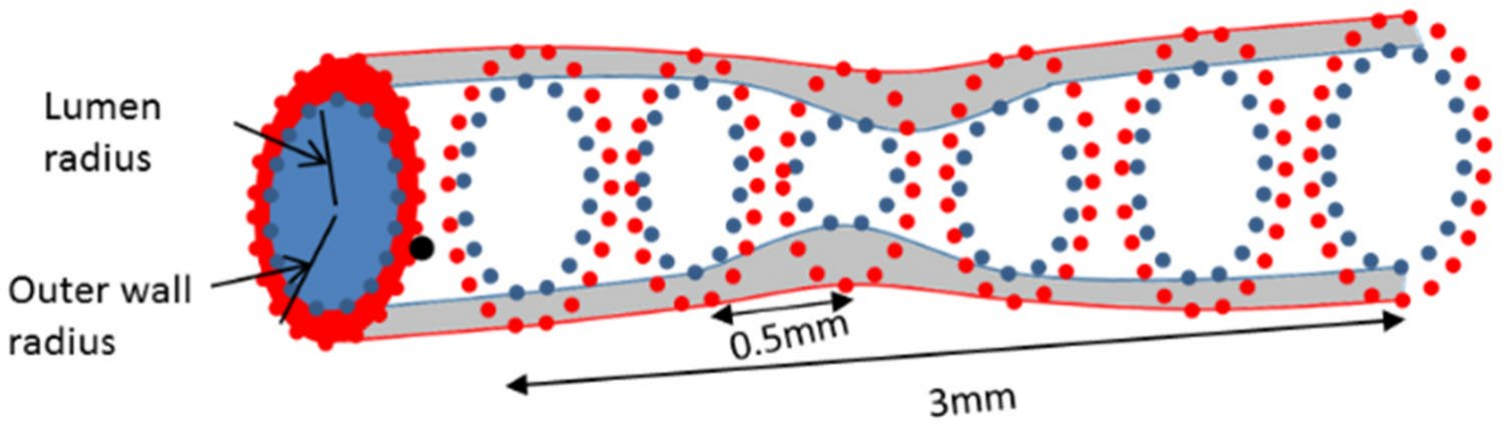
Division of the coronary arteries into 0.5 mm and the combination of six cross sections of 0.5 mm provides 3 mm sub-segments of the artery.

Plaque growth simulations were performed at the baseline reconstructed arteries and we compared the simulated results with the realistic follow-up examination. The validation approach is based on the rationale that the plaque growth model enables the arterial wall deformation in a way that reaches the follow-up geometry as this has been assessed by the CTCA. The validation approach requires the extraction of 0.5 mm—distanced cross-sections of both the baseline and follow-up coronary arteries as shown in Fig. 5. Considering that CTCA has also a mean slice thickness of 0.5 mm, we implemented an approach, proposed by Stone et al.^35^, which is considered good approach for the comparison of computational results with morphological findings from the imaging data or 3D reconstructed arteries. In particular, to eliminate possible registration errors, the rationale is to generate 3 mm segments by combining six sequential 0.5 mm—distanced cross-sections for all geometries (baseline, simulated and follow-up) (Fig. 5).

### Parameters of the plaque growth model

The plaque growth model simulates the major mechanisms of atherosclerosis by employing many differential equations. The equations require some parameters, which in majority are found in literature in experimental studies. All the parameters of the plaque growth model are shown in Table 3.

**Table 3:** The parameters of the plaque growth model.

Parameter	Value
Blood density, *ρ*_*blood*_	1060 kg/m^3^^14^
Blood viscosity, *μ*_*blood*_	0.0035 Pa s^15^
Plasma density, *ρ*_*plasma*_	1000 kg/m^3^^14^
Plasma viscosity, *μ*_*plasma*_	0.001 Pa s^15^
Adventitia pressure of hypertensive patients	30.5 mmHg^36^
Adventitia pressure of normal patients	17.5 mmHg^36^
Partial pressure of oxygen in the arterial wall	60 mmHg^19^
Luminal LDL diffusivity, *D*_*LDL*_, lumen	5 × 10^–12^ m^2^/s^14^
LDL diffusivity in arterial wall, *D*_*LDL*_, wall	8 × 10^–13^ m^2^/s^14^
Luminal HDL diffusivity, *D*_*HDL*_, lumen	5 × 10^–12^ m^2^/s^14^
HDL diffusivity in arterial wall, *D*_*HDL*_, wall	8 × 10^–13^ m^2^/s^14^
Luminal Monocyte diffusivity, *D*_*m*_, lumen	1 × 10^–12^ m^2^/s^37^
Macrophage diffusivity in artery wall, *D*_*m*_, wall	8 × 10^–15^ m^2^/s^38^
OxLDL diffusivity in arterial wall, *D*_*OxLDL*_	8 × 10^–13^ m^2^/s^39^
Cytokine diffusivity in arterial wall, *D*_*S*_	8 × 10^–13^ m^2^/s^38^
Arterial wall porosity, *γ*	0.96^16^
LDL degradation rate, *r*_*LDL*_	1.4 × 10^–4^ s^14^
LDL Solute lag coefficient in arterial wall, *K*_*lag*_	0.1486^14^
Macrophage differentiation rate into foam cells, *k*_*1*_	0.0367 × 10^–4^ m^3^/(cells s)^21,23^
Michaelis–Menten constant for nitric oxide, *K*_*M*_	4.7μΜ^40^
Nitric oxide maximum concentration, *NO*_*max*_	0.585 μmol/(min mg)^40^
**Coefficient of LDL concentration in adventitia**	
For normal patient	0.005^36^
For hypertensive patient	0.015^36^
Darcian permeability, *K*_*w*_	1.2 × 10^–18^ m^2^^14^
Solvent reflection coefficient, *σ*_*f*_	0.997^30^
Macrophage diffusivity in arterial wall, *D*_*M*_	8 × 10^–13^ m^2^/s^38^
Differentiation rate of monocytes into macrophages, *D*_*M*_	1.15 × 10^–6^ s^-1^^22^
Apoptosis rate of monocytes, *m*_*d*_	2.572 s^-1^^41^
OxLDL uptake rate from macrophages, *k*_*2*_	0.12 × 10^-17^m^3^/(s cell)^21^
Cytokine degradation rate, *d*_*c*_	2.3145 × 10^–5^ s^−1^^23^
Cytokine production rate, *d*_*r*_	3.1 × 10^–10^ m^3^/(s cell)^7^
Contractile smooth muscle cell intima concentration	29.26 × 10^12^ cell/m^3^^25^
Synthetic SMCs production coefficient, *S*_*r*_	4.16 × 10^–8^ s^−1^^24^
Cytokine maximum concentration, *c*_*c,max*_	4.2 × 10^9^ mol/m^3^^9^
Collagen secretion rate from synthetic SMCs, *g*_*r*_	2.157 × 10^–11^ g/(s cell)^25^
Collagen degradation rate, *d*_*G*_	3.85 × 10^–7^ s^−1^^26^
Monocyte flow rate coefficient in endothelium, *m*_*r*_	5.5 × 10^–4^ m^3^/(mol day)^9,31^
Endothelium reference wall shear stress value, *WSS0*	1 Pa^9^
Young modulus of isotropic arterial wall	1.06 MPa^42^
Poisson ratio of arterial wall	0.45^42^

### Statistical analysis

All continuous variables are presented as mean ± standard deviation. Wilcoxon, Fisher’s exact and chi-squared tests were used for the comparison of characteristics within group and between groups. Initially, linear regression analysis was performed between all computational variables and the morphological characteristics (lumen area, plaque area, plaque burden and their changes between the baseline and follow-up examination). In the next step, a step-wise multivariate regression model was employed, in which only the associated variables (P < 0.1) were entered.
